# Prevalence of mpox infection among men who have sex with men: a systematic review and meta-analysis

**DOI:** 10.1186/s40249-026-01493-y

**Published:** 2026-08-18

**Authors:** Luqiu Tao, Yanzheng Zou, Lilei Wang, Li Pan, Hongru Zhu, Yili Qian, Yu He, Zhihui Wu, Wei Wang

**Affiliations:** 1 National Health Commission Key Laboratory of Parasitic Disease Control and Prevention, Wuxi, 214064 Jiangsu China; 2 Jiangsu Provincial Key Laboratory on Parasite and Vector Control Technology, Wuxi, 214064 Jiangsu China; 3 Jiangsu Institute of Parasitic Diseases, Wuxi, 214064 Jiangsu China; 4 Shandong Institute of Parasitic Diseases, Shandong First Medical University and Shandong Academy of Medical Sciences, Jining, 272033 Shandong China; 5 Chinese Preventive Medicine Association, Beijing, 100062 China; 6 College of Clinical Medicine for Obstetrics and Gynecology and Pediatrics, Fujian Medical University, Fuzhou, 350001 Fujian China

**Keywords:** Mpox, Men who have sex with men, Prevalence, Meta-analysis

## Abstract

**Background:**

Mpox has been twice declared as public health emergency of international concern by World Health Organization. The 2022–2024 global outbreak has been primarily driven by sexual transmission within men who have sex with men (MSM) networks, predominantly involving Clade IIb, with a subsequent surge of Clade Ib in early 2024. MSM account for a large proportion of confirmed mpox cases. This review aimed to estimate the prevalence of mpox infection among MSM populations.

**Methods:**

We searched PubMed, Web of Science, Embase, China National Knowledge Infrastructure (CNKI) and Wanfang Data for studies published in English or Chinese from inception to 16 December 2024. Eligible studies reported mpox cases among MSM with confirmed diagnosis (laboratory or self-reported) during the outbreak period. Reviews, case reports, and studies without case numbers were excluded. American Agency for Healthcare Research and Quality and Newcastle-Ottawa Scale were adopted for quality assessment. Fixed-effects or random-effects meta-analysis was performed to estimate the pooled prevalence of mpox among MSM. Heterogeneity was measured using Cochran's Q test and *I*^2^ statistics. Meta regression analysis was performed to discover the potential source of the heterogeneity. Funnel plot, Egger's regression test and Begg's rank correlation test were conducted to evaluate the publication bias. Sensitivity analyses were performed by leave-one-out analysis and by stratified validation.

**Results:**

From 1606 retrieved studies, 28 studies were eligible and included, containing a total of 46,598 MSM participants and 1274 mpox cases from 15 countries. The pooled mpox prevalence among MSM population was 2.30% [95% confidence interval (*CI*): 1.29–3.57%]. Meta-regression identified continent as the most significant moderator, with prevalence in South America (9.58, 95% *CI*: 2.71–20.01%) being significantly higher than in Asia (1.14, 95% *CI*: 0.56–1.90%) (*P* = 0.019). Sampling method also contributed to the heterogeneity, whereas no statistically significant difference was observed in mpox prevalence among high-risk MSM, asymptomatic MSM, comparing to the general MSM populations respectively.

**Conclusions:**

This study confirms a significant but highly heterogeneous burden of mpox among MSM worldwide. The findings suggest that the observed prevalence is shaped more by regional epidemiological contexts and study methodologies, which emphasizes the necessity for geographically prioritized interventions and standardizing surveillance to enable accurate risk assessment and guide resource allocation in future outbreaks.

**Graphical Abstract:**

**Figure:**
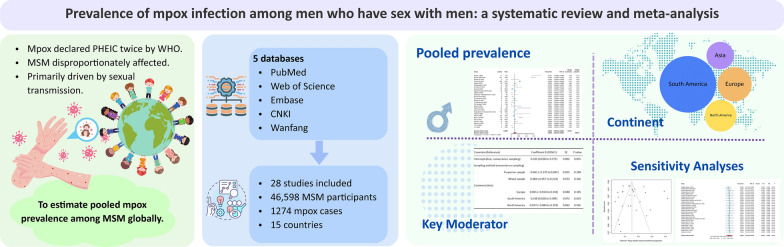

**Supplementary Information:**

The online version contains supplementary material available at 10.1186/s40249-026-01493-y.

## Background

Mpox, previously known as monkeypox, is an infectious zoonotic disease caused by the monkeypox virus. Since the first mpox case reported in Democratic Republic of the Congo in 1970, it has been prevalent sporadically worldwide, with central and west Africa being its main endemic region [1]. In May 2022, many non-endemic countries and regions in Europe and North America reported multiple mpox cases, and according to World Health Organization (WHO) statistics, nearly 100 countries reported over 50,000 confirmed mpox cases and over 280 deaths throughout 2025 [2]. In July 2022, WHO declared the multi-country mpox outbreak a Public Health Emergency of International Concern (PHEIC), as the virus spread rapidly via sexual contact into regions with no prior endemic history [3]. A second PHEIC was issued in August 2024, driven by the emergence of the more transmissible and virulent Clade Ib strain, which expanded from the Democratic Republic of the Congo through sexual networks into East Africa, Europe, and Asia, alongside re-emergence of Clade IIb outbreaks in Nigeria and South Africa [4–6].

Mpox has an incubation period of 5 − 21 days, and generally 6 − 13 days, with symptoms of fever, rashes, lymphadenopathy and muscle ache [7]. Smallpox vaccine can provide a certain degree of cross-protection, hence people who have not received the vaccine are generally susceptible to mpox [8]. Mpox can transmit from human to human via various routes, including respiratory secretions, vertical transmission, direct and indirect contact [9]. During the 2022–2024 global mpox outbreak, sexual contact via infectious lesions on the mucosa and bodily fluids was the major efficient transmission route [9]. While Clade IIb causes less severe disease presentations and has a lower-case fatality rate compared to Clade I, it still imposes a substantial burden due to complications and hospitalizations for severe pain [10–12]. The risk of severe disease and death is particularly pronounced among individuals receiving immunosuppressive treatments or those with untreated or inadequately managed human immunodeficiency virus (HIV) infection [13–15].

Many studies have reported that the population of men who have sex with men (MSM) take up a large proportion in confirmed mpox cases [16–18], and one previous meta-analysis, with its literature search extending to 20 January 2023, noted that approximately 90–100% of confirmed mpox cases globally were disproportionately found among MSM [19]. In addition, high-risk sexual behaviors are considered as the potential risk factors, including chemsex, group sex and multiple sexual partners [20]. The basic reproduction number for mpox transmission among MSM population could be substantially above 1 by fitting to a transmission model with empirical sexual partnership data according to a study from the United Kingdom [21]. Therefore, mpox outbreaks in MSM population may expand quickly due to efficient transmission through their sexual networks, even with a low initial case count [22].

Although the disproportionate burden on MSM is well-recognized, existing prevalence estimates from individual studies show remarkable heterogeneity across different regions, epidemic phases, and study designs. Merely pooling these disparate estimates into a single global prevalence figure without interrogating the sources of heterogeneity may lead to misleading conclusions. To fill the gap in the prevalence of mpox among MSM population, we initiated this systematic review and meta-analysis to synthesize and quantify the status of mpox infection among MSM. Furthermore, our study aims to quantify the potential sources of heterogeneity, and elucidate the epidemiological contexts under which prevalence is likely underestimated, ultimately seeking to provide a refined evidence base for calibrating surveillance systems, prioritizing resources, and designing targeted prevention strategies for MSM populations worldwide.

## Method

### Study design

This systematic review and meta-analysis was conducted following the Preferred Reporting Items for Systematic Reviews and Meta-Analyses (PRISMA) statement [23]. This study has been registered on PROSPERO (International prospective register of systematic reviews) with a registration number of CRD42024587173 in 2024 (available from: https://www.crd.york.ac.uk/PROSPERO/view/CRD42024587173).

### Search strategy

Literature research was conducted in PubMed, Web of Science, Embase, China National Knowledge Infrastructure (CNKI, https://www.cnki.net/) and Wanfang Data (https://www.wanfangdata.com.cn) with the time frame from inception to 16 December 2024. The final research strategy was composed of retrieval words including "monkeypox", "mpox", "MPXV", "MSM", "men who have sex with men" and "gay", and Boolean operators fitting the scope of research. Detailed search strings of each database were listed in Supplementary material 1.

### Inclusion criteria and study selection

The eligibility criteria of the included studies were listed as following: (1) articles were written in English or Chinese; (2) confirmation of mpox diagnosis were mentioned in the study, such as self-reported and laboratory tested; (3) number of mpox cases among MSM population were clearly reported, regardless of whether other population were involved; (4) the research period should be after the confirmation of the first mpox in the study. Case reports, reviews and systematic reviews were excluded.

Retrieved searches were imported to Endnote X9 (Clarivate Analytics; Philadelphia, PA, USA) and duplicate studies were excluded. Two reviewers (Tao L. and Zou Y.) first screened for potential eligible studies based on titles and abstracts. Full texts were assessed for further screening at next stage. All eligible studies were screened and classified according to the eligibility criteria. Discrepancies at every stage were discussed with a third reviewer (Wang W.).

### Data extraction

Data were extracted by two reviewers (Tao L. and Zou Y.) independently and recorded in an Excel table. Any disagreements were discussed with a third reviewer (Wang W.). The data extracted were as follows:

1. Basic information: Author, publication year, study region;
2. Characterization of the study: population characteristic (asymptomatic MSM, high-risk MSM), high risk factors, recruitment source (setting), study period, study design, diagnostic method, sampling method, vaccine-accessibility;
3. Prevalence-related data: sample size, cases;
4. Population characteristics and case definitions:
  Mpox diagnosis: Categorized as laboratory-confirmed (PCR or serology) and self-reported.
  High-risk MSM: Defined variably as MSM living with HIV, using pre-exposure prophylaxis (PrEP), having a recent sexually transmitted infection (STI), or reporting high-risk sexual behaviors (e.g., chemsex, multiple partners).
  Asymptomatic MSM: Defined as participants with no mpox-compatible symptoms at the time of screening, though the specific symptoms systematically assessed differed across studies.
5. Classification of countries followed by Continents and economic status which was based on the World Bank classification;
6. The epidemic phase of each country during the respective study period was based on the mpox case data and epidemic curves provided by WHO (https://ourworldindata.org/mpox);
7. If a study period extended across two calendar years, it was assigned to the initial year;
8. The specific population characteristic was based on the high-risk and asymptomatic definition used in each publication.

### Quality assessment

American Agency for Healthcare Research and Quality (AHRQ) was adopted for quality assessment of cross-sectional studies, which included 11 three-level items (“Yes”/“No”/“Unclear”) [24]. One score would be added for “Yes”, and 0 score for either “No” or “Unclear”. A study scored 8–11 was considered of high quality, 4–7 medium quality, and 1–3 low quality. The Newcastle–Ottawa Scale (NOS; Available from: http://www.ohri.ca/programs/clinical_epidemiology/oxford.asp) assessment was adopted for cohort and case control studies, containing three domains: selection, comparability and outcome. The total score of NOS was 9, with 7–9 indicating high quality, 5–6 medium quality, and 0–4 low quality.

Quality assessment was performed independently by two reviewers (Tao L. and Zou Y.), and disagreements were discussed with a third reviewer (Wang W.).

### Statistical analysis

All data analysis were conducted in R software, version 4.3.2 (R Core Team, Vienna, Austria). Freeman-Tukey double arcsine transformation were adopted for the normality of the prevalence data [25]. Normality of the Freeman-Tukey double arcsine-transformed proportions was assessed using the Shapiro-Wilk test. A fixed-effects or random-effects model was fitted to estimate the pooled prevalence of mpox infection among MSM [26]. To facilitate public health interpretation, absolute risk difference (ARD) with 95% confidence interval (*CI*) was calculated for between-group comparisons [27]. Heterogeneity of the included studies was evaluated by Cochran’s Q test and *I*^2^ statistics, with *I*^2^ values of 25%, 50% and 75% indicating low, moderate and high heterogeneity, respectively [28]. Random-effects model was adopted when the data showed high heterogeneity, otherwise fixed-effect model would be adopted. Univariate meta regression analysis was performed to discover the potential sources of the high heterogeneity. A multivariable meta-regression model was constructed based on univariate results (*P* < 0.1). To evaluate the robustness of the pooled effect estimates, a leave-one-out sensitivity analysis and stratified validation were conducted. Funnel plot, Egger’s regression test and Begg's rank correlation test were adopted to evaluate the publication bias of the included studies. Probabilities reported were two-sided and *P* value < 0.05 was considered statistically significant.

## Results

### Literature search

**Fig. 1:**
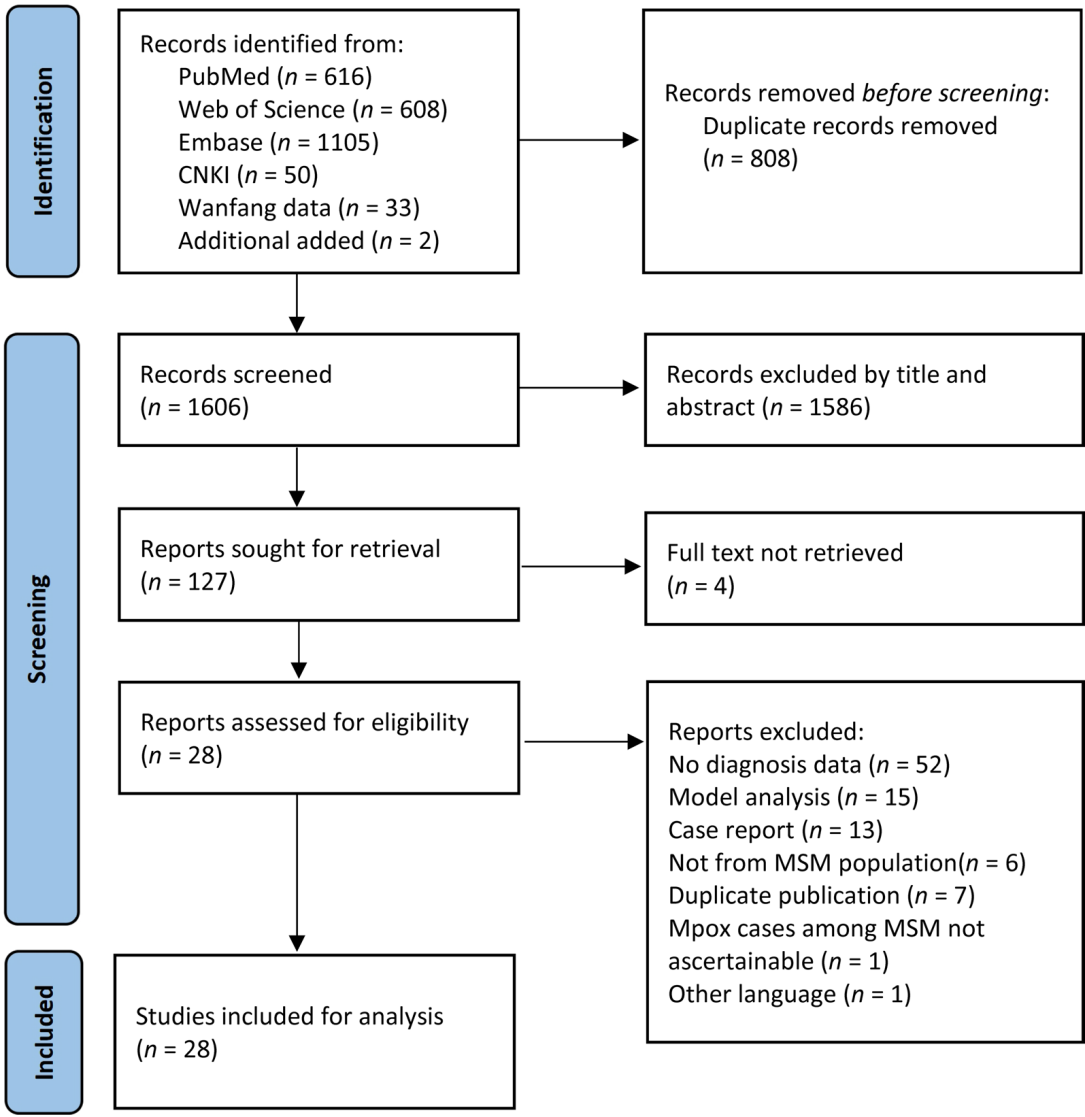
PRISMA flowchart for study selection

Figure 1 presented the flowchart of study screening and selection. A total of 2414 studies were identified, after excluding duplicates, 1606 studies were screened by reading title/abstract, including 1604 searched from databases and 2 added additionally. Of these, 1479 studies were further excluded, and the remaining 127 studies were included for full-text screening. According to the eligibility criteria, 99 studies were further excluded, leaving a total of 28 studies eligible for systematic review and meta-analysis.

### Characteristic of the included studies

Characteristics of the included studies were presented in Table 1. The studies contained a total of 46,598 MSM participants and 1,274 mpox cases from 15 countries, including Austria (*n* = 1), Belgium (*n* = 2), Brazil (*n* = 2), Canada (*n* = 1), China (*n* = 5), Denmark (*n* = 2), France (*n* = 2), Germany (*n* = 2), Ireland (*n* = 1), Israel (*n* = 1), Japan (*n* = 1), Netherlands (*n* = 3), Spain (*n* = 1), UK (*n* = 2) and USA (*n* = 2). All studies were published between 2022 and 2024.

**Table 1:** Characteristics of the included studies on mpox among men who have sex with men

	First author	Publication year	Study region	Sample size	Case	Asymptomatic MSM	High-risk MSM	High risk factor	Recruitment source	Study period	Study design	Diagnose method	Sampling method	Mpox epidemic stage	Accessibility of vaccine	Quality assessment
1	Ghosn J	2024	France	472	77	No	Yes	PrEP	Cohort	May–Sep, 2022	Prospective cohort study	Laboratory test	Convenience	Increase	Accessible	Moderate
2	Santos GRS	2024	Brazil	1452	211	No	No	–	Online	Sep–Dec, 2022	Cross-sectional study	Self-reported	Mixed	Increase	Not reported	Moderate
3	Rossotti R	2023	France	72	0	Yes	Yes	HIV and PrEP	Mixed	Sep–Oct, 2022	Prospective cross-sectional study	Laboratory test	Convenience	Decline	Accessible	Moderate
4	Torres TS	2023	Brazil	5197	292	No	No	–	Online	Oct–Nov, 2022	Cross-sectional study	Self-reported	Convenience	Increase	Unaccessible	Moderate
5	Ogaz D	2023	UK	1333	35	No	No	–	Online	Nov–Dec, 2022	Cross-sectional study	Self-reported	Convenience	Decline	Accessible	Moderate
6	De Baetselier I	2022	Belgium	224	4	No	Yes	HIV and PrEP	Clinic	May, 2022	Cross-sectional study	Laboratory test	Convenience	Not reported	Not reported	Moderate
7	Van Dijck C	2023	Belgium	327	14	No	Yes	HIV and PrEP	Clinic	Jun–Sep, 2022	Retrospective and prospective study	Laboratory test	Convenience	Not reported	Not reported	Moderate
8	Zheng M	2023	China	7538	55	No	No	–	Online	Jul–Aug, 2023	Cross-sectional study	Self-reported	Convenience	Not reported	Unaccessible	Moderate
9	Dukers-Muijrers NHTM	2023	Netherlands	1856	45	No	No	–	Mixed	Jul–Sep, 2022	Cross-sectional study	Self-reported	Convenience	Decline	Accessible	High
10	Gilbert M	2023	Canada	331	0	No	Yes	STI history or high risk sexual behavior	Clinic	Aug, 2022	Cross-sectional study	Self-reported	Convenience	Increase	Accessible	Moderate
11	Grabmeier-Pfistershammer K	2024	Austria	90	0	Yes	Yes	STI	Clinic	Jan–Mar, 2023	Retrospective study	Laboratory test	Convenience	Decline	Accessible	Moderate
12	Mizushima D	2023	Japan	1346	9	Yes	No	–	Cohort	Jan–Mar, 2023	Prospective study	Laboratory test	Convenience	Increase	Unaccessible	High
13	Svartstein AW	2023	Denmark	727	13	No	Yes	HIV	Cohort	May–Oct, 2022	Prospective cohort study	Laboratory test	Convenience	Increase	Accessible	Moderate
14	Minhaj FS	2023	USA	225	3	Yes	No	–	Clinic	Jun–Sep, 2022	Cross-sectional serologic study	Laboratory test	Convenience	Increase	Accessible	Moderate
15	Thomassen SE	2024	Denmark	224	1	Yes	Yes	PrEP	Clinic	Aug–Oct, 2022	Prospective cross-sectional study	Laboratory test	Convenience	During	Accessible	Moderate
16	Pitt-Kendall R	2023	UK	1159	2	No	No	–	Clinic	Aug–Oct, 2022	Retrospective cross-sectional study	Laboratory test	Purposive	Decline	Not reported	Moderate
17	Pestel J	2022	Germany	53	0	Yes	Yes	HIV and PrEP	Clinic	Jun–Jul, 2022	Cross-sectional study	Self-reported	Purposive	Increase	Accessible	Moderate
18	Pathela P	2024	USA	166	13	Yes	No	–	Clinic	Jul–Sep, 2022	Cross-sectional Serological study	Laboratory test	Convenience	Peak	Accessible	Moderate
19	Adam PCG	2024	Netherlands	2460	73	No	No	–	Online	Jul–Aug, 2022	Cross-sectional study	Self-reported	Convenience	Decline	Accessible	Moderate
20	Siegenbeek van Heukelom ML	2023	Netherlands	7306	135	No	No	–	Clinic	May–Sep, 2022	Case control study	Laboratory test	Convenience	Including the peak	Accessible	Moderate
21	Agustí C	2023	Spain	113	7	Yes	Yes	HIV or PrEP or high-risk sexual behavior	Online	Aug–Oct, 2022	Cross-sectional study	Laboratory test	Convenience	Not reported	Not reported	Moderate
22	Chen F	2024	China	7725	127	No	No	–	Online	Aug, 2023	Cross-sectional study	Self-reported	Purposive	Increase	Unaccessible	Moderate
23	Marcus U	2024	Germany	1119	69	No	No	–	Clinic	Apr–Jul, 2023	Cross-sectional study	Laboratory test	Convenience	Decline	Accessible	High
24	Gao Y	2024	China	2403	56	No	No	–	Online	Oct, 2023–Mar, 2024	Cross-sectional study	Self-reported	Mixed	Decline	Unaccessible	High
25	Poliker E	2024	Israel	665	19	No	No	–	Online	Sep–Oct, 2022	Cross-sectional study	Self-reported	Convenience	Decline	Accessible	Moderate
26	Gilmore J	2024	Ireland	163	5	No	No	–	Mixed	Dec, 2022–Jan, 2023	Cross-sectional study	Self-reported	Convenience	Decline	Accessible	Moderate
27	Tian Y	2024	China	562	1	No	No	–	Mixed	Sep, 2023	Cross-sectional study	Laboratory test	Convenience	Increase	Unaccessible	High
28	Li J	2024	China	1290	8	No	No	–	Clinic	Aug, 2023	Cross-sectional study	Self-reported	Convenience	Increase	Unaccessible	Moderate

*MSM* men who have sex with men, *PrEP* pre-exposure prophylaxis, S*TI* sexually transmitted infection, *HIV* human immunodeficiency virus, – not applicable

MSM participants were mainly recruited through online social media (*n* = 9), sexual health clinics (*n* = 12), MSM-related cohort (*n* = 3) and mixed sources (*n* = 4). Eight studies focused on asymptomatic MSM and 10 studies focused on high-risk MSM who were living with HIV, using PrEP, with STIs or having high-risk sexual behaviors. All studies adopted non-probability sampling methods when recruiting participants, of which 23 using convenience sampling, 3 using purposive sampling and 2 using mixed sampling methods containing convenience and snowball sampling. Nine studies were conducted during the emerging phase of the mpox outbreak, 14 studies were conducted during the declining phase and 5 studies covered the whole outbreak. Among 23 studies that mentioned vaccine accessibility, vaccine was accessible in 16 studies while inaccessible in the other 7 when the studies were performed. In 15 studies, mpox cases were confirmed through laboratory testing, while diagnoses in the other 13 studies were derived from self-reported information. Geographically, the included studies were conducted in Europe (*n* = 16), Asia (*n* = 7), North America (*n* = 3), and South America (*n* = 2). The majority of studies (*n* = 21) were from high income countries, while the remaining 7 studies were from upper middle-income countries.

### Quality of included studies

We evaluated the quality of the 28 included studies using AHRQ and NOS assessment. A total of 23 studies were of medium quality (20 from AHRQ and 3 from NOS) and 5 studies (all from AHRQ) were of high quality (Supplementary material 2).

### Results of meta-analysis

#### Pooled prevalence of mpox among MSM

**Fig. 2:**
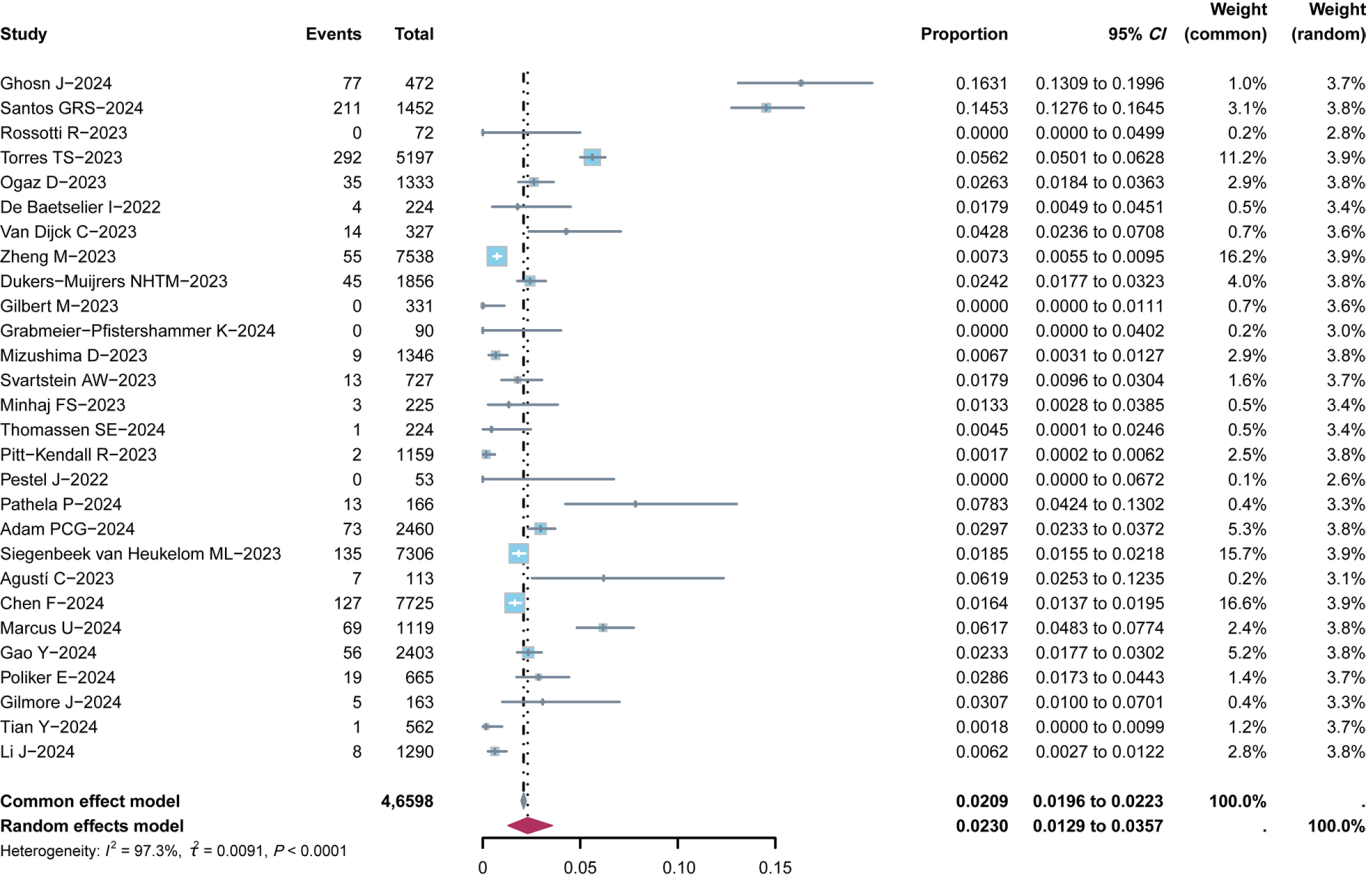
Forest plot of the prevalence of mpox infection among men who have sex with men

The Shapiro–Wilk test confirmed that the double arcsine-transformed proportions did not significantly deviate from normality (*W* = 0.936, *P* = 0.087). The mpox prevalence among MSM ranged from 0.00 to 16.31% in the included studies. Significant heterogeneity was found across the studies (*I*^2^ = 97.3%, *τ*^2^ = 0.0091, *P* < 0.0001). Based on the random-effects model, the pooled prevalence of mpox infection among MSM populations was 2.30% (95% *CI*: 1.29–3.57%) (Fig. 2).

#### Subgroup analysis

Among different recruited MSM populations, the prevalence of mpox infection in high-risk MSM was 1.88% (95%* CI*: 0.22–4.73%), lower than that in MSM recruited from general populations (2.50, 95% *CI*: 1.37–3.94%) but with no statistically significant difference (*P* = 0.79). The ARD between high-risk MSM and general population was − 0.62% (95% *CI*: − 1.14 to − 0.10%). Asymptomatic MSM (1.28, 95% *CI*: 0.10–3.33%) had lower mpox infection prevalence than general MSM population (2.71, 95% *CI*: 1.44–4.35%), with no statistically significant difference (*P* = 0.34). The prevalence of mpox among MSM recruited from different settings was not significantly different (*P* = 0.23), with 4.36% in cohort population, 3.68% in online population, 1.40% in clinic population, and 1.08% in mixed recruitment settings, respectively. The prevalence of mpox among MSM in different continents was statistically significant difference (*P* = 0.02), with 9.58% in South America, 2.40% in Europe, 1.87% in North America and 1.14% in Asia. The ARD was 8.4% (95% *CI*: 7.7–9.2%) between South America and Asia, 7.2% (95% *CI*: 6.4–7.9%) between South America and Europe, and 1.3% (95% *CI*: 1.0–1.5%) between Europe and Asia. No statistically significant differences in mpox prevalence among MSM were observed across subgroups stratified by World Bank classification (*P* = 0.91), mpox epidemic stage (*P* = 0.11), vaccine accessibility (*P* = 0.25), sampling methods (*P* = 0.09), or diagnostic methods (*P* = 0.96). Mpox prevalence among MSM in studies performed in 2022 (2.89, 95% *CI*: 1.49–4.69%) was higher than in studies performed in 2023 (1.21, 95% *CI*: 0.37–2.46%), with no statistically significant difference (*P* = 0.06) (Table 2). Mpox prevalence among MSM in studies of high quality (1.91, 95% *CI*: 0.49–4.20%) was lower than in those of moderate quality (2.40, 95% *CI*: 1.22–3.94%), with no statistically significant difference (*P* = 0.06).

**Table 2:** Subgroup analysis and meta regression of Mpox prevalence among men who have sex with men

Variables	Studies (*n*)	Sample size	Cases	Subgroup analysis					Meta regression		
				Prevalence (%)	95% *CI* (%)	*I*^2^ (%)	*τ*^2^	Between-group* P* value	*R*^2^ (%)	*Q*_m_	*P*
Population								0.79	0.00	0.07	0.80
High-risk MSM	10	2633	116	1.88	0.22 to 4.73	94.7	0.0139				
General MSM	18	43,965	1158	2.50	1.37 to 3.94	98.0	0.0074				
Symptom								0.34	0.00	0.79	0.37
Asymptomatic MSM	8	2289	33	1.28	0.10 to 3.33	81.7	0.0068				
General MSM	20	44,309	1241	2.71	1.44 to 4.35	98.0	0.0098				
Setting								0.23	3.23	4.13	0.25
Cohort	3	2545	99	4.36	0.00 to 16.12	98.7	0.0315				
Online	9	28,886	875	3.68	1.75 to 6.26	98.8	0.0079				
Clinic	12	12,514	249	1.40	0.42 to 2.85	93.0	0.0062				
Mixed	4	2653	51	1.08	0.04 to 3.06	86.8	0.0036				
Continent								0.02	22.24	9.41	0.02
Europe	16	17,698	480	2.40	1.11 to 4.11	94.3	0.0081				
Asia	7	21,529	275	1.14	0.56 to 1.90	91.8	0.0016				
South America	2	6649	503	9.58	2.71 to 20.01	99.0	0.0114				
North America	3	722	16	1.87	0.00 to 8.18	93.4	0.0161				
World Bank classification								0.91	0.00	0.02	0.89
High income	21	20,431	524	2.22	1.13 to 3.62	93.8	0.0079				
Upper middle income	7	26,167	750	2.57	0.52 to 6.07	99.1	0.0138				
Mpox epidemic stage								0.11	6.81	3.90	0.14
Emerging phase	9	21,525	291	1.17	0.50 to 2.07	91.9	0.0023				
Declining phase	14	16,177	742	2.46	1.00 to 4.48	97.3	0.0099				
Whole outbreak	5	8896	241	4.62	0.99 to 10.59	97.4	0.0161				
Vaccine-accessibility								0.25	2.46	2.62	0.27
Yes	16	17,262	488	2.33	1.03 to 4.07	93.6	0.0087				
No	7	26,061	548	1.38	0.48 to 2.71	98.7	0.0039				
Not reported	5	3275	238	4.18	0.69 to 10.16	98.3	0.0169				
Sampling method								0.09	10.69	4.98	0.08
Convenience sample	23	33,806	878	2.27	1.26 to 3.56	96.4	0.0076				
Purposive sample	2	8937	129	0.45	0.00 to 1.89	92.9	0.0281				
Mixed sample	3	3855	267	7.22	0.13 to 23.42	99.5	0.0023				
Diagnostic methods								0.96	0.00	0.00	0.97
Laboratory test	15	14,132	348	2.24	0.83 to 4.24	95.5	0.0109				
Self-report	13	32,466	926	2.38	1.06 to 4.17	98.3	0.0079				
Research performed year								0.06	6.09	2.55	0.11
2022	20	24,525	949	2.89	1.49 to 4.69	97.1	0.0103				
2023	8	22,073	325	1.21	0.37 to 2.46	95.4	0.0041				
Quality								0.06	0.00	1.17	0.56
Moderate	23	39,312	1094	2.40	1.22 to 3.94	97.6	0.0101				
High	5	7286	180	1.91	0.49 to 4.20	95.7	0.0057				

*MSM* men who have sex with men

### Meta regression

Univariable meta-regressions were performed to explore potential sources of heterogeneity (Table 3). The covariate continent explained the largest proportion of heterogeneity (*R*^2^ = 22.24%, *P* = 0.02), with studies from South America reporting significantly higher prevalence than those from Asia. Sampling method also accounted for 10.69% of variance (*R*^2^ = 10.69%, *P* = 0.08). All other covariates, including population, symptom, World Bank classification, diagnostic methods and quality had *R*^2^ values of 0.00% and showed no significant association (all *P* > 0.10). According to multi-variables meta-regression, the model was statistically significant (*Q*_M_ = 12.53, df = 5, *P* = 0.028) and jointly accounted for 24.81% of the between-study heterogeneity. After adjusting for sampling method, the mpox prevalence among MSM in South America remained significantly higher than in Asia (β = 0.168, 95% *CI*: 0.028–0.309, *P* = 0.019). The coefficients for sampling method were not statistically significant in the adjusted model.

**Table 3:** Results of the multi-variables meta regression analysis

Covariate (reference)	Coefficient β (95% *CI*)	SE	*P* value
Intercept (Asia, convenience sampling)	0.105 (0.038 to 0.173)	0.002	0.035
Sampling method (convenience sampling)			
Purposive sample	− 0.061 (− 0.170 to 0.047)	0.055	0.268
Mixed sample	0.083 (− 0.057 to 0.224)	0.072	0.246
Continent (Asia)			
Europe	0.065 (− 0.014 to 0.143)	0.040	0.105
South America	0.168 (0.028 to 0.309)	0.072	0.019
North America	0.037 (− 0.084 to 0.159)	0.062	0.545

### Publication bias and sensitivity analysis

**Fig. 3:**
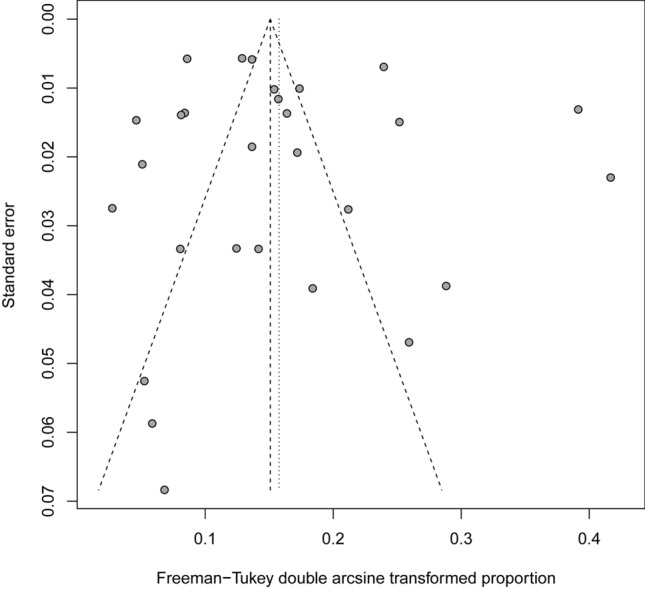
Funnel plot of publication bias on mpox studies

Publication bias was assessed using multiple complementary methods. Funnel plot for studies published on mpox among MSM presented symmetric (Fig. 3) and the result of Egger’s regression test indicated no significant publication bias (β = 1.17, SE = 2.02, *P* = 0.57). This finding was corroborated by Begg's rank correlation test, which also showed no evidence of funnel plot asymmetry (*z* = 0.41, *P* = 0.68).

**Fig. 4:**
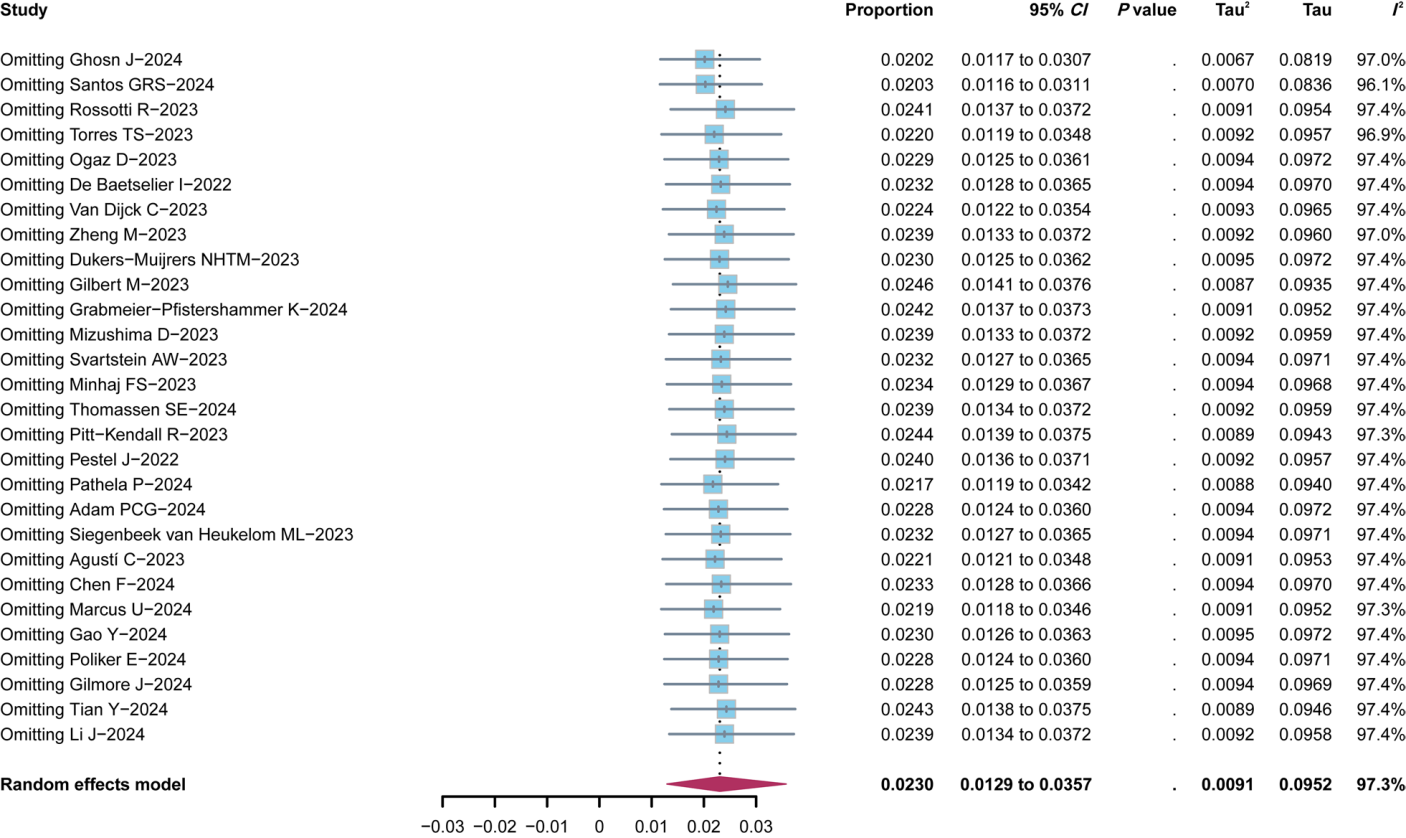
“Leave-one-out” sensitivity analysis of the included studies

“Leave-one-out” sensitivity analysis indicated no significant impact of individual studies, affirming the robustness and reliability of the results (Fig. 4). When stratified by study quality, the between-group difference was not statistically significant (Q = 0.28, *P* = 0.60), and the confidence intervals overlapped substantially. A sensitivity analysis restricted to high-quality studies alone yielded a pooled prevalence of 1.91% (95% *CI*: 0.49–4.20%), which was consistent with the overall estimate of 2.30% (95% *CI*: 1.29–3.57%), indicating that the inclusion of moderate quality studies did not materially distort the overall findings. When stratified by diagnostic method, the between-group difference was not statistically significant (Q = 0.00, *P* = 0.96), and the confidence intervals overlapped substantially. A sensitivity analysis restricted to laboratory-confirmed studies alone yielded a pooled prevalence of 2.24% (95% *CI*: 0.83–4.24%), which was consistent with the overall estimate of 2.30% (95% *CI*: 1.29–3.57%), indicating that diagnostic method heterogeneity did not materially bias the pooled prevalence estimate.

## Discussion

In this research, we systematically reviewed the prevalence of mpox among the MSM population from a total of 28 included studies. The results showed that the pooled prevalence was 2.30%, ranging from 0 to 16.31% among all the included researches. In addition, the prevalence of mpox among MSM was significantly different among four continents, with higher prevalence in South America (9.58%).

Our findings showed that academic reports on mpox prevalence among MSM populations were first published in 2022. This finding suggested that although mpox infection had long been endemic, its epidemiological characteristics diverged significantly from previous patterns since the global outbreak in 2022 [29]. Surveillance data revealed that the majority of confirmed cases occurred among adults aged 21 − 55 years in MSM populations, with close contact being the predominant transmission route [30]. There was limited research focusing on the mpox prevalence among MSM population. One research which estimated the burden of mpox among MSM in South Africa reported that with a large number of undetected cases, the case ascertainment rate ranged from 1 to 8% [31], which was in line with the pooled prevalence of 2.30% in our research.

Globally, cases of mpox peaked in August 2022 [32]. In our study, the mpox prevalence was higher in researches performed in 2022 and during the whole outbreak, which were consistent with global epidemiological trends. According to the surveillance data from WHO, the Region of the Americas had 11% mpox confirmed cases which was higher than other regions from January 1 to June 22, 2022 [32]. The monthly data provided by WHO also showed that cases in Region of the Americas were more than European Region and Western Pacific Region [32]. This geographical disparity must also be contextualized within the socio-demographic movements of the period. The years 2022 − 2023 witnessed substantial migration flows from Central to North America [33]. Overcrowding, limited access to healthcare, and fear of seeking services due to immigration status could have facilitated local mpox transmission and simultaneously created significant barriers to case detection and reporting within this mobile population. Within the broader Americas region, we identified the prevalence of mpox among MSM in South America significantly exceeding that of Asia, which may be driven by region-specific factors such as dense sexual network connectivity, delays in the rollout of targeted public health interventions, or regional variations in vaccine accessibility during the critical early outbreak period [34]. The ARD translated the observed relative disparities into concrete public health metrics. The excess of 84 cases per 1,000 MSM in South America, compared with Asia represents a substantial burden that underscores the urgency of geographically prioritized interventions in specific South American settings, mainly in Brazil.

The prevalence of mpox among MSM in different studies varied greatly. Among them, two invesitgations reported the highest mpox prevalence of 16.31% and 14.53%, which might be attribute to active and high-risk sexual behaviors among participants [35, 36]. Many surveillance reports and studies have indicated that high-risk populations accounted for a large proportion of mpox-positive cases, including those who were living with HIV, using PrEP, with STIs or having high-risk sexual behaviors [11, 20, 37–39]. From a biological susceptibility perspective, HIV-associated immunosuppression may compromise both innate and adaptive immune responses, potentially increasing vulnerability to mpox acquisition and severe disease. Individuals co-infected with HIV and mpox are more susceptible to secondary bacterial infections, and symptoms such as rash, proctitis, and diarrhea are more prevalent, with higher case fatality rates compared to mpox infection alone [40]. Similarly, STI-related mucosal inflammation and disruption of epithelial barriers could facilitate viral entry during sexual contact, further elevating biological susceptibility [41]. However, in our study, we found slightly lower prevalence in high-risk MSM, which warranted interpretation from the dual perspectives of biological susceptibility and structural vulnerability. Studies of general MSM populations often employ broad online or community-based recruitment, which may capture a wider spectrum of individuals, including those with undiagnosed or asymptomatic infections. In contrast, researches focusing on high-risk MSM typically recruit from clinical settings where frequent testing is the norm. This leads to earlier case detection and reporting, potentially resulting in a lower prevalence at the time of a cross-sectional survey within the high-risk cohort. Prevention paradox may also explain this phenomenon: individuals identified as 'high-risk' are often more engaged with sexual health services [42]. This engagement, in turn, may confer higher health literacy, better access to prevention tools like early mpox vaccination, and more frequent testing behaviors. Their enhanced prevention awareness and proactive health-seeking could mitigate their baseline behavioral risk. Among the 10 studies focusing on high-risk MSM in our analysis, 7 were conducted in countries where mpox vaccines were accessible, and participants were predominantly recruited from clinical cohorts with established healthcare linkages. Their enhanced prevention awareness and proactive health-seeking behaviors may have partially mitigated their baseline behavioral risk. Nevertheless, it is important to emphasize that our subgroup analysis remains ecological in nature: individual-level data on HIV viral load, CD4 count, STI type, and vaccination timing were not available across studies. The interplay between biological susceptibility and structural vulnerability proposed here should therefore be viewed as hypothesis-generating, requiring validation through future studies with individual-level clinical and behavioral covariates.

In addition, asymptomatic individuals may contribute to mpox transmission [43]. The mpox prevalence among asymptomatic MSM was relatively lower in our study comparing to the general MSM populations with no significant difference. The low pooled prevalence observed in our study aligns with findings from other focused screening studies. For instance, Armenteros-Yeguas et al. [44] reported a prevalence of 0.3% among asymptomatic MSM in a Spanish cross-sectional study, and Hampel et al. [45] identified 2 cases among 201 asymptomatic PrEP users (1%). And several studies also screened no detected asymptomatic cases among high-risk populations [46, 47]. However, a critical methodological nuance must be emphasized: several individuals in those studies who tested positive subsequently developed mpox-related symptoms [44, 45]. This indicates that some cases captured in "asymptomatic" screenings were likely in the pre-symptomatic phase of infection. Consequently, single-time-point cross-sectional studies are inherently limited in their ability to distinguish true asymptomatic infection from pre-symptomatic states. Individuals tested outside a narrow virological window would be misclassified, leading to a systematic underestimation of the true reservoir of infection that can transmit the virus prior to or in the absence of overt clinical disease. Passive, symptom-based reporting systems are inherently insensitive to pre-symptomatic and asymptomatic transmission, which indicates the imperative for integrating active serial testing in high-risk network studies and developing validated serological assays to better quantify the true infection reservoir and transmission dynamics. It can be acknowledged that the absence of characteristic symptoms can lead to ignoring prevention and treatment, potentially increasing mpox spread. This highlights the importance of combined national surveillance, clinical monitoring, and enhanced public awareness to strengthen mpox prevention and control.

Currently, four vaccines, ACAM2000, MVA‐BN, LC16m8, and OrthopoxVac are approved for mpox and smallpox prevention and more than 40 mpox vaccine candidates are in development [48]. Many studies have reviewed the acceptance of mpox vaccination previously, and the MSM population showed the highest acceptance and uptake rate [49–51]. However, there exists a remarkable gap between the intention and overall uptake of mpox vaccination globally, which can be explained by variations of the study timing and the accessibility of the mpox vaccine [51]. In this research, the results indicated that the prevalence of mpox among MSM was slightly higher in countries where mpox vaccines were available. This might be explained by several factors. First, depending on the local epidemic dynamics and transmission intensity, some countries provided vaccination for high-risk population after the beginning of the mpox outbreaks. Therefore, the high prevalence reflected the epidemic dynamics before the widespread uptake of vaccines. Second, due to the limited vaccine accessibility and eligibility criteria, the real uptake rate among MSM may have been insufficient, leading to limited protection effects. Additionally, countries where vaccines were accessible typically had more robust public health surveillance system, resulting in higher detection rates of mpox cases. Finally, while European and American nations were among the first to promote mpox vaccination, they also have more densely connected and sexually active MSM community networks, which may have facilitated transmission.

The implication of this study lies in its comprehensive, phase-specific, and systematic research of the disease burden of mpox prevalence among MSM. High-risk MSM, including who were living with HIV, using PrEP, with STI diseases and having high-risk sexual behavior, should acquire comprehensive knowledge about mpox and check regularly both for themselves and their sexual partners. Reducing number of sexual partners and avoiding high-risk sexual behaviors can reduce infection risk especially during the start phase and outbreak stage of a mpox epidemic. In addition, early detection and accurate differentiation of mpox from chickenpox, measles, syphilis, and other STIs are critical to ensure timely treatment. High-risk populations should receive mpox vaccination as soon as eligible. Targeted digital outreach is crucial to engage the broader and less clinically connected MSM population. Furthermore, every individual can be infected with mpox although the MSM population was affected greatly. Thus, discrimination and stigma against mpox must be prohibited, as these may undermine disease prevention and control efforts by interfering with seeking treatment and prevention services among high-risk populations, including trans people, MSM and gender diverse communities.

Several limitations of this study should be acknowledged when interpreting the findings. First, our meta-analysis is inherently susceptible to the ecological fallacy. The higher prevalence in South America represents correlations at the population or study level and should not be directly equated with individual-level risk factors. Second, despite our multivariable model explaining a notable proportion of heterogeneity, substantial residual heterogeneity still remains high. This indicates that the model could not account for several variables, including granular sexual network structures, precise local vaccination timelines, community stigma levels, and nuanced epidemic phases, may have biased the observed associations and explain part of the substantial residual heterogeneity. Third, the geographical distribution of included studies was markedly uneven. Only two studies were available from South America, both conducted in Brazil; no studies were identified from Africa or Oceania. Consequent, the pooled estimate for South America cannot be considered representative of the entire continent, and the between-continent comparison should be interpreted with caution. The significant difference detected between South America and Asia in our meta-regression is driven entirely by Brazilian data and may reflect country-specific epidemiological dynamics rather than a broader continental pattern. Fourth, significant methodological heterogeneity across primary studies, particularly in the case definitions and population characteristic, poses challenges for direct comparison. Specifically, inconsistency in diagnostic criteria (laboratory-confirmed versus self-reported) may introduce information bias. Although our subgroup and sensitivity analyses found no statistically significant differences by diagnostic method or population characteristic, the overlapping confidence intervals in certain strata may partly reflect the limited number of studies in each category and do not rule out the possibility of non-differential misclassification bias. Fifth, we were unable to examine the impact of mpox virus clade on the pooled prevalence due to the lack of related information. Given that our literature search concluded in December 2024, our synthesis primarily reflects the epidemiology of the Clade IIb-driven pandemic phase (2022 − 2024 mpox outbreak). The emergent spread of Clade Ib since early 2024, which is associated with different transmission dynamics and severity profiles, necessitates cautious extrapolation of our conclusions to the evolving present and future of the mpox outbreak. Future surveillance studies should routinely report viral clade information to enable quantitative clade-stratified analyses. Sixth, HIV prevalence, while reported in a subset of studies, was not uniformly documented, precluding a formal meta-regression. These limitations constrain our ability to elucidate the underlying causes of regional prevalence disparities and highlight the need for standardized reporting of these variables in future mpox surveillance research. Finally, the ARD reported in this study is derived from pooled subgroup proportions and should be interpreted as ecological contrasts at the study level rather than individual-level risk differences.

## Conclusions

Our systematic review and meta-analysis provide a comprehensive overview of mpox prevalence in the MSM population. We identify geographical disparities, particularly in South America, suggesting that the epidemic burden is highly uneven. Our findings mainly reflect the Clade IIb mpox pandemic phase, indicating that targeted interventions must be informed by localized epidemiological surveillance and adaptable to the evolving variants.

## Supplementary Information

Supplementary Material 1.

Supplementary Material 2.

## Data Availability

All data presented in this study are available upon request by contact with the corresponding author.

## Abbreviations

AHRQ: American Agency for Healthcare Research and Quality
ARD: Absolute risk difference
*CI*: Confidence interval
CNKI: China National Knowledge Infrastructure
HIV: Human immunodeficiency virus
MSM: Men who have sex with men MSM
NOS: Newcastle-Ottawa Scale
PHEIC: Public Health Emergency of International Concern
PrEP: Pre-exposure prophylaxis
PRISMA: Preferred Reporting Items for Systematic Reviews and Meta-Analyses
STI: Sexually transmitted infection
WHO: World Health Organization
