# Supplementary material for: Prevalence of mpox infection among men who have sex with men: a systematic review and meta-analysis
Source: Infect Dis Poverty. 2026 Aug 18;15:89. doi: 10.1186/s40249-026-01493-y (PMC13483694; doi:10.1186/s40249-026-01493-y)
Supplement: Supplementary file 1 [file 40249_2026_1493_MOESM1_ESM.docx]

**Searching strategy** (Search string used on 16/12/2024):

**PubMed:**

| **Number** | **Domain** | **Search terms** |
| --- | --- | --- |
| 1 | Mpox | (monkeypox) OR (Mpox) OR (MPXV) |
| 2 | MSM | (MSM) OR ("men who have sex with men") OR (gay) |
| **Searching strategy:** 1 AND 2 | | |

**Web of Science:**

| **Number** | **Domain** | **Search terms** |
| --- | --- | --- |
| 1 | Mpox | TI=(monkeypox) OR AB=(monkeypox)) OR (TI=(mpox) OR AB=(mpox)) OR (TI=(MPXV) OR AB=(MPXV)) |
| 2 | MSM | (TI=(MSM) OR AB=(MSM)) OR (TI=("men who have sex with men") OR AB=("men who have sex with men")) OR (TI=(gay) OR AB=(gay)) |
| **Searching strategy:** 1 AND 2 | | |

**Embase:**

| **Number** | **Domain** | **Search terms** |
| --- | --- | --- |
| 1 | Mpox | ('monkeypox'/exp OR monkeypox OR mpxv OR mpox) |
| 2 | MSM | ('men who have sex with men' OR msm OR gay) |
| **Searching strategy:** 1 AND 2 | | |

**CNKI**

| **Number** | **Domain** | **Search terms** |
| --- | --- | --- |
| 1 | Mpox | TKA='猴痘' |
| 2 | MSM | TKA='男男' OR TKA='msm' |
| **Searching strategy:** 1 AND 2 | | |

**Wanfang:**

| **Number** | **Domain** | **Search terms** |
| --- | --- | --- |
| 1 | Mpox | 题名或关键词:(猴痘) |
| 2 | MSM | 题名或关键词:(MSM or 男男) |
| **Searching strategy:** 1 AND 2 | | |
